# Association between physical activity, peak expiratory flow, and cognitive function in aging: a cross-sectional analysis

**DOI:** 10.1186/s12877-024-05080-4

**Published:** 2024-05-26

**Authors:** Bin Dong, Yang Yue, Zhe Wang, Min Sun, Yuehui Wang

**Affiliations:** 1 Department of Geriatrics, Jilin Geriatrics Clinical Research Center, The First Hospital of Jilin University, Changchun, 130021 China; 2 School of Education, Changchun Normal University, Changchun, China

**Keywords:** Aging, Cognitive function, Peak expiratory flow, Physical activity

## Abstract

**Background:**

The aging global population is experiencing escalating challenges related to cognitive deficits and dementia. This study explored the interplay between pulmonary function, physical activity, and cognitive function in older U.S. adults to identify modifiable risk factors for cognitive decline.

**Methods:**

Utilizing NHANES 2011–2012 data, we conducted a cross-sectional analysis of 729 participants aged ≥ 60 years. Cognitive function, peak expiratory flow (PEF), and physical activity were assessed. Weighted logistic regression and mediation analyses were employed to examine associations.

**Results:**

The sample size was 729 (weighted mean [SD] age, 67.1 [5.3] years; 53.6% female participants). Preliminary correlation analysis indicated a positive correlation between the global cognitive score and physical activity (β = 0.16; *p* < 0.001), recreational activity (β = 0.22; *p* < 0.001), and PEF in percent predicted (PEF%) (β = 0.18; *p* < 0.001). Compared to those with a PEF% >100%, the PEF% (80-100%) group (OR, 2.66; 95% CI, 1.34–5.29; *p* = 0.005) and PEF% <80% group (OR, 3.36; 95% CI, 1.67–6.76; *p* = 0.001) were significantly associated with higher cognitive deficits risk. Recreational activity meeting guidelines was linked to a lower risk of cognitive deficits (OR, 0.24; 95% CI, 0.10–0.57; *p* = 0.001). Mediation analysis demonstrated that PEF mediates the relationship between physical activity and cognitive function.

**Conclusion:**

This study revealed significant associations between lower PEF, diminished physical activity, and increased cognitive deficits in elderly individuals. The results supported the hypothesis that pulmonary function may mediate the connection between activity and cognitive health, emphasizing the importance of respiratory health in cognitive aging. Recognizing these associations is crucial for clinical care and public health policy aiming to mitigate cognitive decline in aging populations. While these findings are intriguing, validation through longitudinal design studies is deemed necessary.

**Supplementary Information:**

The online version contains supplementary material available at 10.1186/s12877-024-05080-4.

## Introduction

As the global population ages, the challenges posed by cognitive deficits and dementia have become increasingly prominent in healthcare and public health. Given the current absence of effective treatments, there is an urgent need to identify modifiable risk factors contributing to cognitive decline [1, 2], thereby facilitating the development of effective preventive measures.

Emerging evidence indicates a connection between pulmonary function and cognitive performance [3–5]. A decline in pulmonary function typically commences around the age of 25 [5], and nonsmoking adults experience an average annual decline of 1% [6]. It is widely believed that impaired pulmonary function may impact the central nervous system through pathways such as inflammation-mediated vascular diseases, fibrinolysis dysfunction, oxidative stress, and changes in neurotransmitter metabolism caused by hypoxia [7, 8]. Various studies have demonstrated associations between different pulmonary function indicators and cognitive function [3, 9–14]. In a community-based cohort study with a 27-year follow-up period, participants experiencing midlife lung disease and impaired pulmonary function exhibited a 58% higher risk of developing dementia or mild cognitive impairment in later life [15]. Additionally, two separate studies have reported that a diagnosis of COPD is associated with an approximately 80% increased risk of experiencing mild cognitive impairment within a 5-year period and developing dementia within a 25-year period, respectively [16, 17]. Moreover, in cross-sectional analyses, poorer pulmonary function has been associated with a smaller brain volume and a greater burden of white matter hyperintensity [18]. Therefore, enhancing pulmonary function may have meaningful implications for cognitive function, irrespective of whether individuals have pulmonary disease.

Furthermore, studies have suggested that elevated levels of physical activity are correlated with a diminished risk of cognitive decline [19, 20] and dementia, including Alzheimer’s disease [21]. Additionally, an association between prolonged sitting and poorer cognitive performance has been noted, although further investigation is required to understand the relationship between sedentary time and overall dementia risk [22–24]. Current research generally supports the idea that lifestyle modifications, such as increasing physical activity levels [25–27], can contribute to maintaining or enhancing respiratory function. According to a longitudinal study of aging in Canada, the physical activity patterns of older adults were linked to respiratory function, irrespective of smoking status [26]. Furthermore, high levels of physical activity may delay declines in respiratory function [27] and potentially decelerate cognitive decline [15].

We conducted this study to investigate the relationship between physical activity, pulmonary function, and cognitive function in older adults in the U.S. Given the observational and cross-sectional nature of the data, causality cannot be inferred. Nevertheless, by exploring the associations between exercise, pulmonary function, and cognitive deficits, we aimed to provide valuable insights for the prevention of cognitive deficits.

## Methods

### Participants

National Health and Nutrition Examination Survey (NHANES) is a series of cross-sectional, multi-stage, continuous surveys conducted by the National Center for Health Statistics (NCHS) in the United States. Its primary goal is to assess the health and nutritional status of the U.S. population. The NHANES employs a complex sampling design that takes into account participants’ age, race/ethnicity, and geographic location to obtain a representative sample of the United States’ non-institutionalized population. The research protocol received approval from the National Center for Health Statistics Ethics Review Board, and all participants provided written informed consent.

This study is a cross-sectional analysis utilizing data from the 2011–2012 cycle, including data on cognitive performance, pulmonary function, and physical activity. Following the NHANES analysis guidelines [28], the data were weighted to represent the corresponding U.S. civilian population aged ≥ 60 years (eAppendix, eFigure in the Supplement). This study strictly followed the guidelines provided by the Strengthening the Reporting of Observational Studies in Epidemiology (STROBE).

### Cognitive function assessment

The NHANES study comprises three tests assessing cognitive function. Detailed information can be found online [29] and in the eAppendix of the Supplement. In this study, we standardized the scores for each test (CERAD, AFT, and DSST) and then summed them to obtain a global cognitive score, reflecting cognitive function. We assessed potential clinical relevance by defining the presence of at least mild cognitive deficits as having total composite scores less than 1 standard deviation (SD) below the mean [30–32].

### Peak expiratory flow (PEF) in percent predicted

Peak expiratory flow (PEF) is a physiological parameter initially proposed for estimating airflow obstruction. Additionally, the PEF in percent predicted (PEF%) proved to be a more accurate and convenient strategy for predicting impaired pulmonary function in older adults [33]. The PEF% is the ratio of the measured PEF to its predicted value. The optimal threshold for detecting airflow limitation is when the PEF% reaches 80% [34]. Jackson et al. analyzed data from the NHANES III and defined a PEF% < 80% as abnormal [35]. In this study, participants were categorized into three groups based on PEF%: < 80%, 80 − 100%, and > 100%.

### Physical activity

According to the 2008 Federal Guidelines for Physical Activity [36], we reflected participants’ physical activity by the total exercise time within 7 days, categorized into four levels: No physical activity; Below guideline: moderate physical activity (< 150 min/week), vigorous physical activity (< 75 min/week); Meet guideline: moderate physical activity (150 min/week-300 min/week), vigorous physical activity (75 min/week-150 min/week); Exceed guideline: moderate physical activity (> 300 min/week), vigorous physical activity (> 150 min/week). In this study, physical activity encompasses both work-related and recreational activities, offering a comprehensive measure of individuals’ daily activity levels, regardless of the specific activities involved. The work activity group includes participants who did not engage in recreational activities beyond the Below Guideline level. The recreational activity group includes participants who did not engage in work activity beyond the Below Guideline level.

### Covariates

Considering the potential confounding factors among PEF, cognition, and physical activity, we simultaneously investigated several covariates. We enhanced the model fit by including several covariates in the model: age, gender, race/ethnicity, education level, body mass index, smoking history, lung disease, heart disease, hypertension, stroke, and diabetes. These covariates were self-reported by participants or their proxies during NHANES interviews. Age was treated as a continuous variable. Gender was considered a binary variable: male or female. Since widely accepted lung capacity reference values for Asian Americans and other ethnicities are currently lacking, race/ethnicity was limited to three groups: non-Hispanic Whites, non-Hispanic Blacks, and Mexican Americans. Education level was categorized as less than high school, high school, some college, and college degree or higher. Body mass index (BMI) was calculated based on self-reported height and weight. Smoking status included never smoked, former smoker, and current smoker. History of lung disease, heart disease, hypertension, stroke, or diabetes was classified as a binary variable: yes or no.

### Statistical analysis

According to the NHANES analysis tutorial, considering the complex sampling design, we applied sampling weights to correct for all the statistical analyses, ensuring that the results possessed national representativeness. We initially conducted a comprehensive case analysis, excluding all participants with missing or invalid data on cognitive performance, pulmonary function, physical activity, and covariates. Continuous variables were described using weighted means (SD), while categorical variables were presented as weighted frequencies (%). The global cognitive score, representing overall cognitive function and following a normal distribution, was obtained by summing the standardized scores of CERAD, AFT, and DSST. Cognitive deficits (yes or no) as a categorical variable were defined as a global cognitive score less than 1 SD below the mean. Missing values for categorical variables were treated as a separate category for comparison.

Spearman correlation analysis was employed in this study to examine the relationships between variables. Weighted logistic regression models were utilized to analyze the associations between PEF and physical activity, respectively, with cognitive deficits. The strength of the associations was expressed as odds ratios (OR) and 95% confidence intervals (95% CI). The mediation analysis involved one independent variable (physical activity/ recreational activity/work activity), one dependent variable (global cognitive score), and one mediating variable (PEF%). Given the multi-categorical nature of the independent variable, relative and overall mediation analysis methods were employed based on previous research [37]. Data analysis was conducted using SPSS 26.0 software, with the PROCESS Macro performing Bootstrap-based mediation effect tests and estimating 95% confidence intervals. The number of bootstrap samples was set at 5000. Control variables, including age, gender, race, and education, were introduced into the model. If the confidence interval included 0, it indicated no significant indirect effect at a 5% significance level.

All the statistical tests were conducted as two-sided, with statistical significance defined at a p-value < 0.05. The analyses were performed using IBM SPSS Statistics version 26.0 for Windows and Stata version 16.0.

## Results

### Demographics and baseline characteristics

The distribution of participants, as weighted, is presented in Table 1. A total of 729 participants aged 60 years and older were included in the analysis. The mean (SD) age was 67.1 (5.3) years. Among them, 46.4% (weighted proportion) were male. Cognitive deficits were identified in 34.0% (*n* = 248) of participants. In comparison with participants classified as having cognitive deficits, those with better cognitive function were more likely to be female, possessed higher educational attainment, demonstrated a higher level of PEF%, and engaged in a greater amount of physical activity, particularly recreational activities. Participants classified with cognitive deficits exhibited a significantly higher likelihood of having a history of diabetes, hypertension, coronary heart disease, and stroke.

**Table 1:** Weighted characteristics of the study populations, NHANES 2011 to 2012

Characteristic		Cognitive deficits	
	Total	NO	YES
	(*n* = 729)	(*n* = 481)	(*n* = 248)
Age, mean (SD), y	67.1(5.3)	66.6(4.9)	69.3(6.0)
Gender, weighted %			
Male	46.4	43.5	57.9
Female	53.6	56.5	42.1
Race, weighted %			
Mexican American	3.0	1.8	8.0
Non-Hispanic white	87.1	91.4	70.2
Non-Hispanic black	9.9	6.8	21.8
Education, weighted %			
< High school	14.0	7.2	41.2
High school	21.4	18.9	31.2
Some college	29.1	32.5	15.5
College degree or more	35.5	41.4	12.1
BMI, mean (SD), kg/m^2^	29.4(6.2)	29.4(6.1)	29.6(6.8)
Physical activity, weighted %			
No physical activity	32.3	28.3	48.2
Below guideline	14.7	15.0	13.3
Meet guideline	17.6	19.7	9.2
Exceed guideline	35.4	37.0	29.3
Recreational activity, weighted %			
No recreational activity	50.2	44.7	72.0
Below guideline	14.1	14.8	11.1
Meet guideline	16.8	19.7	5.3
Exceed guideline	18.9	20.8	11.6
Work activity, weighted %			
No work activity	58.5	56.8	65.1
Below guideline	11.4	12.5	6.8
Meet guideline	8.8	9.6	5.8
Exceed guideline	21.3	21.0	22.3
Cognitive score, mean (SD)			
CERAD	26.0(5.8)	27.8(4.6)	19.1(4.7)
AFT	19.4(6.0)	20.9(5.5)	13.3(3.5)
DSST	56.7(16.1)	61.7(13.0)	36.6(10.8)
Global cognitive score	1.0(2.4)	1.8(1. 8)	-2.3(1.2)
PEF in percent predicted, mean (SD)	1.0(0.24)	1.1(0.22)	1.0(0.25)
PEF in percent predicted group, weighted %			
> 100%	58.7	63.7	39.0
80–100%	26.8	24.9	34.2
< 80%	14.5	11.4	26.8
Smoking, weighted %			
Never	48.8	50.8	40.5
Former	38.7	38.3	40.1
Current	12.6	10.8	19.4
Lung disease, weighted %	20.5	21.3	17.2
High blood pressure, weighted %	72.2	69.6	82.7
Diabetes, weighted %	17.6	13.7	32.7
Cardiovascular diseases, weighted %	10.3	9.7	12.8
Stroke, weighted %	3.9	3.0	7.5

Notes: Data presented are mean (SD), or (%). SD, Standard Deviation; BMI, body mass index; PEF, peak expiratory flow; CERAD, the word learning and recall modules from the Consortium to Establish a Registry for Alzheimer’s disease; AFT, Animal Fluency Test; DSST, Digit Symbol Substitution Test; Global cognitive score was obtained by summing the standardized scores of CERAD, AFT, and DSST; Cognitive deficits (yes or no) were defined as a global cognitive score less than 1 standard deviation below the mean

### Preliminary correlation analyses

The correlation analysis between variables were analyzed via Spearman correlation analysis and are summarized in Table 2. The global cognitive score was positively correlated with physical activity (β = 0.16; *p* < 0.001), recreational activity (β = 0.22; *p* < 0.001), as well as PEF% (β = 0.18; *p* < 0.001). Furthermore, PEF% demonstrated a significant correlation with physical activity (β = 0.10; *p* < 0.01) and recreational activity (β = 0.19; *p* < 0.001). However, the correlation between global cognitive score, PEF%, and work activity was not found to be significant. In addition, a noteworthy negative correlation was identified between age and the global cognitive score.

**Table 2:** Correlation coefficient matrix of variables

Variables	1	2	3	4	5	6	7	8
1. Age								
2. Gender	0.003							
3. Race	-0.05	0.02						
4. Education	-0.09^*^	0.03	-0.10^**^					
5. PEF in percent predicted	0.03	0.11^**^	-0.02	0.14^***^				
6. Physical activity	-0.05	-0.12^**^	-0.09^*^	0.14^***^	0.10^**^			
7. Recreational activity	-0.06	-0.07	-0.08^*^	0.23^***^	0.19^***^	0.66^***^		
8. Work activity	-0.01	-0.07	-0.07	0.02	0.01	0.72^***^	0.17^***^	
9. Global cognitive score	-0.26^***^	0.21^***^	-0.19^***^	0.55^***^	0.18^***^	0.16^***^	0.22^***^	0.06

Notes: ^*^p < 0 0.05; ^**^p < 0 0.01; ^***^p < 0 0.001

### Logistic regression analysis

The prevalence of cognitive deficits varied among PEF percent predicted groups, with rates of 26.8% (*n* = 64), 34.2% (*n* = 67), and 39.0% (*n* = 117) (Table 1). The occurrence of cognitive deficits was lower in groups with higher PEF% than in those with lower PEF%.

The relationship between PEF% and cognitive deficits, evaluated through weighted logistic regression, is presented in Table 3. In Model 1, adjusted for age, gender, race, and education level, the risk of having cognitive deficits was greater in the PEF% (80-100%) and PEF% <80% groups compared to the PEF% >100% group. Specifically, the PEF% <80% group (OR, 3.45; 95% CI, 1.67–7.12) had a significantly greater risk.

**Table 3:** Weighted logistic regression analysis of PEF and recreational activity in relation to cognitive deficits

	Odds Ratios and 95% Confidence Intervals		
	Model 1	Model 2	Model 3
PEF%			
> 100%	reference	reference	reference
80–100%	2.65(1.37,5.12) *p* = 0.004	2.74(1.40,5.37) *p* = 0.003	2.66(1.34,5.29) *p* = 0.005
< 80%	3.45(1.67,7.12) *p* = 0.001	3.60(1.79,7.25) *p* < 0.001	3.36(1.67,6.76) *p* = 0.001
Recreational activity			
No activity	reference	reference	reference
Below guideline	0.64(0.30,1.39) *p* = 0.263	0.63(0.29,1.38) *p* = 0.251	0.764(0.34,1.70) *p* = 0.51
Meet guideline	0.24(0.10,0.57) *p* = 0.001	0.22(0.10,0.54) *p* = 0.001	0.24(0.10,0.57) *p* = 0.001
Exceed guideline	0.50(0.19,1.29) *p* = 0.15	0.48(0.19,1.26) *p* = 0.138	0.55(0.21,1.46) *p* = 0.23

Notes: Model 1 adjusts for age, gender, race, and education. Model 2 includes Model 1 covariates plus BMI, physical activity, history of smoking and lung disease. Model 3 includes Model 2 covariates plus history of high blood pressure, cardiovascular diseases, diabetes, and stroke; PEF%, PEF in percent predicted

In models 2 and 3, additional adjustments were made for BMI, smoking, physical activity, lung disease, heart disease, hypertension, stroke, and diabetes risk factors. The results from Models 2 and 3 were similar to those from Model 1, indicating that, compared to those in the > 100% PEF group, the 80-100% and < 80% PEF groups exhibited a greater risk of cognitive deficits (Table 3). Model 3, which included all the covariates, revealed a significantly greater risk in the PEF% (80-100%) group (OR, 2.66; 95% CI, 1.34–5.29) and in the PEF% <80% group (OR, 3.36; 95% CI, 1.67–6.76).

The relationships between recreational activity and cognitive deficits, evaluated through weighted logistic regression, are also presented in Table 3. In Model 1, adjusted for age, gender, race, and education level, compared to the group with no recreational activity, the group with recreational activity time meeting recommended levels showed a lower risk of cognitive deficits (OR, 0.24; 95% CI, 0.10–0.57), while there were no significant differences for recreational activity time below or above the recommended levels. The results in Model 3, including all covariates, were consistent with those in Model 1, indicating that compared to not engaging in recreational activity, engaging in recreational activity time meeting recommended levels was statistically associated with a lower risk of cognitive deficits (OR, 0.24; 95% CI, 0.10–0.57), with statistical significance.

However, there was no relationship between physical activity and cognitive deficits, as shown in eTable 1 of the Supplement.

### Mediation analyses

Mediation analyses with physical activity and recreational activity as independent variables are presented in eTable 2 of the Supplement and Table 4, respectively. Taking the “no physical activity” group as a reference, in the “Exceed guideline” group, the significance of the indirect impact of physical activity through PEF% was confirmed (95% bootstrap CI = 0.001–0.096). A bootstrapped 95% CI confirmed that the indirect impact of physical activity, mediated by PEF%, was 0.039 on the global cognitive score. Furthermore, the indirect effect of PEF contributed to 7.1% of the total variance in global cognitive scores.

**Table 4:** Mediating model examination by bootstrap

	Indirect effect				Direct effect			
	Effect	SE	LL 95%CI	UL 95%CI	Effect	SE	LL 95%CI	UL 95%CI
Physical activity → Global cognitive score								
Below guideline	0.034	0.025	-0.008	0.089	0.224	0.212	-0.192	0.639
Meet guideline	0.037	0.027	-0.008	0.098	0.740	0.226	0.296	1.184
Exceed guideline	0.039	0.024	0.001	0.096	0.511	0.168	0.181	0.840
Recreational activity → Global cognitive score								
Below guideline	0.046	0.028	0.003	0.111	0.150	0.208	-0.259	0.558
Meet guideline	0.075	0.037	0.013	0.157	0.701	0.219	0.271	1.132
Exceed guideline	0.067	0.034	0.011	0.146	0.448	0.204	0.049	0.848

Notes: SE, standard error; CI, Confidence Intervals

In comparison to the “no recreational activity” group, the recreational activity time in the “meet guideline” group showed a significant indirect effect through PEF% (95% bootstrap CI = 0.013–0.157). PEF% influenced global cognitive score with an indirect effect of 0.075 and accounted for 9.7% of the total variance in global cognitive score. In the “Exceed guideline” group, recreational activity also demonstrated a significant indirect effect through PEF% (95% bootstrap CI = 0.011–0.164). PEF%, as a mediating factor, impacted the global cognitive score with an indirect effect of 0.067 and contributed to 13.0% of the total variance in the global cognitive score.

**Fig. 1:**
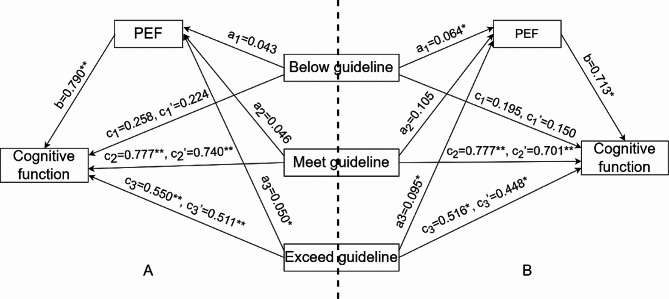
Proposed models that investigate mediated effects. Notes: The results of the relative mediation analysis are based on the reference level of “No activity”; (**A**) Proposed models that investigate mediated effects in the association between physical activity and cognitive function; (**B**) Proposed models that investigate mediated effects in the association between recreational activity and cognitive function; ^*^P < 0.05, ^**^P < 0.01, ^***^P < 0.001

These findings confirmed our hypothesis that PEF might serve as a mediating factor in the association between activity and cognitive function. Figure 1 illustrates the mediation model along with standardized path coefficients.

## Discussion

This study explored the association between physical activity, pulmonary expiratory flow (PEF), and cognitive function in individuals aged 60 years and older in the U.S. Additionally, the mediating role of PEF in the relationship between physical activity and cognitive function was investigated. The findings revealed that higher levels of physical activity, particularly in recreational activities, and a higher percentage of predicted PEF were associated with fewer cognitive deficits. Furthermore, this study found that PEF mediates the relationship between physical activity and cognitive function, although mediation analysis using longitudinal designs would provide more compelling evidence. The results suggested that interventions to improve PEF in older adults and enhance recreational activity might directly or indirectly reduce the risk of cognitive deficits in the elderly.

Previous studies have indicated that PEF serves not only as an indicator of pulmonary function but also as a general marker of health, particularly in the elderly population. The PEF has been associated with chronic conditions in older individuals, such as dementia, muscle atrophy, and frailty, among others [30, 38, 39]. A study utilizing data from the National Health and Aging Trends Study (NHATS) investigated the correlation between PEF and incident dementia in 5935 older adults from 2011 to 2014 [30]. The findings suggested that PEF might be considered a potentially modifiable risk factor for dementia, with higher PEF categories providing greater protection against incident dementia. Consistent with prior research, the present study revealed a significant association between PEF and cognitive function, with a lower PEF% associated with a higher risk of cognitive impairment. The research further provided supporting evidence, indicating that even after adjusting for multiple confounding factors, including age, gender, race, education, smoking, and medical history, the risk of cognitive deficits remained statistically significant when the PEF% decreased to less than 80%. This study might represent the first instance of analyzing the relationship between PEF and cognitive function using data provided by the NHANES database.

However, the pathophysiological mechanisms through which impaired pulmonary function affects cognitive function remain unclear. The prevailing viewpoint suggests that a decline in pulmonary function leads to inadequate brain oxygenation, resulting in neuronal damage and subsequently manifesting as a decline in cognitive ability [40–42]. Additionally, there is an alternative perspective proposing a close association between oxygen deficiency-induced inflammation, oxidative stress, and the progression of brain aging [43, 44]. A study on the neurocognitive assessment of chronic obstructive pulmonary disease (COPD) has shown that cognitive impairment is more severe in COPD patients than in healthy controls [45]. In the present study, after excluding 142 participants with pulmonary diseases such as chronic bronchitis, emphysema, or asthma, the direct relationship between PEF% and the global cognitive score remained significant (*p* < 0.001, eTable 3 in the Supplement).

Physical activity (PA) has long been acknowledged as a widely accepted measure for reducing the risk of age-related cognitive decline. However, due to inconsistencies in the parameters of physical activity used in different studies, variations in cognitive measurement methods, and inconsistent assessments of moderating factors, the dose-response effects of physical activity on cognition and brain health remain unclear [46–48]. In the present study, after we adjusted for demographic factors, lifestyle variables, and medical history, we obtained interesting findings. Compared to individuals not engaging in recreational activities, the risk of cognitive deficits was lower in the group meeting the recommended exercise duration, with no statistically significant differences between groups with exercise durations below or exceeding the recommended guidelines. Extended or excessive exercise may result in insufficient recovery time, accompanied by physical stress and the release of cortisol [49], collectively posing potential adverse effects on cognitive functions. Additionally, the study revealed that the duration of work activity was not significantly correlated with cognitive function, a result not previously reported in the literature. The analysis suggested that the disparity in cognitive impact between recreational and work activities stems from the multifaceted nature of the former, embracing diverse experiences, social interactions, and positive emotions. In contrast, the latter, characterized by repetitive tasks and limited social engagement, correlates with inferior emotional experiences, thereby attenuating the cognitive benefits of physical activity.

Physical activity not only enhances cognitive function in older adults but also has been proven effective in safeguarding and improving pulmonary function [26, 27]. Our hypothesis posited that pulmonary function could act as a mediator in the association between physical activity and cognitive function. Further exploration of the associations between physical activity, pulmonary function, and cognitive function may contribute to clarifying causal pathways involved. Mediation analysis demonstrated that PEF served as a mediator in the correlation between physical activity, particularly recreational activity, and cognitive function. This suggested that older adults with poorer pulmonary function might face a greater risk of cognitive impairment when physical activity is reduced. PEF had mediating effects on 9.7% and 13.0% of the variance in the relationship between recommended and exceeded recommended recreational activity time and cognitive function, respectively. This finding suggested that sufficient recreational activity time could not only directly reduce the risk of cognitive impairment but also indirectly reduce the risk through mediating effects.

### Limitations

This study has several limitations. First, the pulmonary reference values used in this study were based on samples from the NHANES III covering individuals aged 8 to 80 years in the U.S. However, due to the racial/ethnic of NHANES III individuals classifications, Hankinson et al.‘s research only provided pulmonary measurement reference values for non-Hispanic white, non-Hispanic black, and Mexican American populations [50], lacking reference values for other Hispanic, Asian American, and other ethnic groups. The analysis focused solely on U.S. non-Hispanic white, non-Hispanic black, and Mexican American populations, which might not represent the entire elderly population in the U.S. Second, within the NHANES study, pulmonary function, cognitive performance, and physical activity were simultaneously collected for only the 2011–2012 cycle. Thus, the study relied exclusively on the data from this specific cycle, potentially impacting the accuracy and reliability of the findings. Third, due to the cross-sectional study design and potential residual (unmeasured) confounding factors, the causal relationships were not established in this study. Future research should employ longitudinal designs or clinical trials to further assess the associations among physical activity, pulmonary function, and cognitive function. Forth, it’s important to note that the thresholds for work and recreational activities may differ depending on the intensity and duration of the activities involved. However, it is regrettable that we have not uncovered any studies addressing the question of whether the thresholds for work-related physical activity and recreational activity are consistent. In this study, we initially investigate the influence of diverse activity types on cognitive function, adhering to the conventional 150/75 minutes guideline. We hope this serves as a reference for future research delving deeper into the relationship between different types of activities and health outcomes. Fifth, although comparative analysis of basic characteristic data of participants included and not included in this study (eTable 4 in the Supplement) showed intergroup differences in several variables, the complexity of the sampling design and weight adjustments, coupled with the use of multiple logistic regression analysis to control confounding factors, enhanced the persuasiveness of the results and ensured sample representativeness.

## Conclusion

The present study revealed an association in the American elderly population aged older than 60 years, where higher PEF and a longer duration of physical activity, especially recreational activity, were correlated with a reduced risk of cognitive impairment. This research represents the first attempt to evaluate the mediating influence of PEF on the connection between physical activity and cognitive function in older adults. These findings contribute to a better understanding of the interplay among physical activity, pulmonary function, and cognitive function. Importantly, these findings hold significance for the future prevention, treatment, and care of cognitive deficits in the elderly.

### Electronic supplementary material

Below is the link to the electronic supplementary material.

Supplementary Material 1

## Data Availability

The data that support the findings of this study are openly available at: https://www.cdc.gov/nchs/nhanes/index.htm.

## Abbreviations

PEF: peak expiratory flow
PEF%: PEF in percent predicted
NHANES: National Health and Nutrition Examination Survey
