# Supplementary material for: Association between physical activity, peak expiratory flow, and cognitive function in aging: a cross-sectional analysis
Source: BMC Geriatr. 2024 May 26;24:460. doi: 10.1186/s12877-024-05080-4 (PMC11129504; doi:10.1186/s12877-024-05080-4)
Supplement: Supplementary file 1 — Supplementary Material 1 [file 12877_2024_5080_MOESM1_ESM.docx]

**Supplementary Online Content**

**eAppendix.** Analytic Sample Size, Peak Expiratory Flow (PEF) Measures, Physical Activity Measures, and Cognition Measures

**eFigure**. Flowchart Illustrating How We Arrived at the Analytic Samples

**eTable 1**. Weighted logistic regression analysis between physical activity and cognitive deficits.

**eTable 2**. Mediating effect of PEF in percent predicted between activity and Global cognitive score.

**eTable 3**. Correlation Coefficient Matrix of Variables in people without pulmonary diseases.

**eTable 4**. Comparison of the characteristics between participants included and excluded in this study.

**eReferences**

**eAppendix.** Analytic Sample Size, Peak Expiratory Flow (PEF) Measures, Physical Activity Measures, and Cognition Measures

1. Analytic Sample Size: In the NHANES database, cognitive performance data, lung function data, and physical activity data were only collected simultaneously during the 2011-2012 cycle. Therefore, this study only analyzed data from the 2011-2012 cycle. In total, 9,756 individuals participated in NHANES during the 2011-2012 period. Participants aged 60 and above were eligible for our study, as cognitive assessments were only conducted within this age range. The lung measurement reference values used in this study, based on the NHANES III sample, only provided lung measurement reference values for non-Hispanic Whites, non-Hispanic Blacks, and Mexican Americans, lacking reference values for other Hispanic groups, Asian Americans, and other ethnicities.[1] Thus, participants of other Hispanic groups, Asian Americans, other ethnicities, and those below the age of 60 were initially excluded. Subsequently, individuals with missing cognitive function, lung function, and other data were further excluded, resulting in a final sample of 729 individuals aged 60 or above (**eFigure**).
2. Peak Expiratory Flow (PEF) Measures: NHANES conducted lung function assessments on eligible participants aged 6 to 79 during three survey cycles from 2007 to 2012. Detailed inclusion and exclusion criteria are available online[2]. This study utilized data from the first baseline lung function measurement only, adhering to NHANES official guidance by including data with quality grades A and B. Peak Expiratory Flow (PEF) is a physiological measurement initially proposed for estimating airflow obstruction. The predicted PEF values are calculated using a formula established by HANKINSON et al. for analyzing the NHANES III spirometry data [1]. The formula is as follows: lung function parameter=b_0_+b_1_*age+b_2_*age^2^+b_3_*height^2^. The parameters b0, b1, b2, and b3 are associated with age, race, and gender, and can be referenced in the literature by HANKINSON et al. Additionally, PEF in percent predicted proved to be a more accurate and convenient strategy for predicting impaired pulmonary function in older adults[3].
3. Physical Activity Measures: Physical activity was measured using the Global Physical Activity Questionnaire (GPAQ).[4] Each participant was required to complete a survey regarding physical activities performed in the previous 7 days, including the intensity, duration, and frequency of physical activities. In the NHANES study, Physical Activity encompasses both work activity and recreational activity. Work activity includes paid or unpaid work, studying or training, household chores, yard work, brisk walking, or carrying light loads. Recreational activity refers to sports, fitness, bicycling, swimming, or golf. Vigorous physical activity refers to activities that require strenuous physical effort and result in a significant increase in breathing or heart rate, while moderate physical activity refers to activities that require moderate physical effort and lead to a slight increase in breathing or heart rate.
4. Cognition Measures: NHANES comprises three cognitive function assessment tests, namely, the word learning and recall modules from the Consortium to Establish a Registry for Alzheimer’s disease (CERAD), the Animal Fluency Test (AFT), and the Digit Symbol Substitution Test (DSST). CERAD includes CERAD-WL and CERAD-DR, evaluating participants' immediate and delayed memory for new language information.[5] In the continuous three-phase learning stage of CERAD-WL, participants are instructed to loudly read 10 unrelated words and then recall them as much as possible. Following the completion of the other two cognitive tests (AFT and DSST), the CERAD-DR test is conducted, where participants are asked to recall the words from CERAD-WL. In this study, we aggregate the scores of CERAD-WL and CERAD-DR tests to obtain the CERAD total score. AFT is a language fluency task.[6] In the practice test phase, participants are asked to name three pieces of clothing to ensure smooth participation. Subsequently, participants are required to verbally list as many animal names as possible within one minute, with the correct number constituting their final score. DSST is utilized to assess the subject's attention (sustained attention), information processing speed, and working memory.[7] Participants receive a paper with a grid containing 9 numbers and corresponding symbols at the top, below which are 133 boxes with numbers. Participants are then tasked with writing down as many matching symbols as possible within two minutes. The DSST score is the total number of correct matches.

**eFigure. Flowchart Illustrating How We Arrived at the Analytic Samples**

| **eTable 1. Weighted logistic regression analysis between physical activity and cognitive deficits.** | | | |
| --- | --- | --- | --- |
|  | Odds Ratios and 95% Confidence Intervals | | |
|  | Model 1 | Model 2 | Model 3 |
| Physical activity |  |  |  |
| No physical activity | reference | reference | reference |
| Below guideline | 0.609(0.282,1.317) p=0.208 | 0.606(0.28,1.311) p=0.203 | 0.707(0.323,1.545) p=0.385 |
| Meet guideline | 0.408(0.146,1.137) p=0.086 | 0.408(0.144,1.152) p=0.09 | 0.429(0.15,1.221) p=0.113 |
| Exceed guideline | 0.57(0.289,1.124) p=0.105 | 0.573(0.288,1.142) p=0.113 | 0.641(0.326,1.257) p=0.195 |
| Notes: Model 1 adjusts for age, gender, race, and education. Model 2 includes Model 1 covariates plus BMI, history of smoking and lung disease. Model 3 includes Model 2 covariates plus history of high blood pressure, cardiovascular diseases, diabetes, and stroke. | | | |

| **eTable 2. Mediating effect of PEF in percent predicted between activity and Global cognitive score.** | | | | | | | | | | | | | | | |
| --- | --- | --- | --- | --- | --- | --- | --- | --- | --- | --- | --- | --- | --- | --- | --- |
|  | Model 1 | | | | | Model 2 | | | | | Model 3 | | | | |
|  | B | SE | β | t | p | B | SE | β | t | p | B | SE | β | t | p |
| Physical activity | | | | | | | | | | | | | | | |
| Age | 0.001 | 0.002 | 0.026 | 0.698 | 0.486 | -0.100 | 0.013 | -0.227 | -7.824 | <0.001 | -0.101 | 0.013 | -0.229 | -7.931 | <0.001 |
| Gender | 0.063 | 0.019 | 0.125 | 3.385 | <0.001 | 1.031 | 0.141 | 0.213 | 7.327 | <0.001 | 0.981 | 0.141 | 0.203 | 6.953 | <0.001 |
| Race | -0.012 | 0.011 | -0.040 | -1.103 | 0.270 | -0.164 | 0.080 | -0.059 | -2.042 | 0.042 | -0.155 | 0.080 | -0.056 | -1.935 | 0.053 |
| Education | 0.028 | 0.009 | 0.122 | 3.256 | 0.001 | 1.094 | 0.066 | 0.493 | 16.658 | <0.001 | 1.072 | 0.066 | 0.483 | 16.278 | <0.001 |
| No physical activity | reference | reference | reference | reference | reference | reference | reference | reference | reference | reference | reference | reference | reference | reference | reference |
| Below guideline | 0.043 | 0.028 | 0.169 | 1.524 | 0.128 | 0.258 | 0.212 | 0.106 | 1.214 | 0.225 | 0.224 | 0.212 | 0.092 | 1.058 | 0.290 |
| Meet guideline | 0.046 | 0.030 | 0.185 | 1.556 | 0.120 | 0.777 | 0.227 | 0.320 | 3.427 | <0.001 | 0.740 | 0.226 | 0.305 | 3.275 | 0.001 |
| Exceed guideline | 0.050 | 0.022 | 0.197 | 2.243 | 0.025 | 0.550 | 0.168 | 0.227 | 3.270 | 0.001 | 0.511 | 0.168 | 0.211 | 3.040 | 0.002 |
| PEF% | NA | NA | NA | NA | NA | NA | NA | NA | NA | NA | 0.790 | 0.282 | 0.082 | 2.803 | 0.005 |
| Recreational activity | | | | | | | | | | | | | | | |
| Age | 0.001 | 0.002 | 0.031 | 0.848 | 0.397 | -0.099 | 0.013 | -0.224 | -7.730 | <0.001 | -0.100 | 0.013 | -0.226 | -7.833 | <0.001 |
| Gender | 0.064 | 0.018 | 0.127 | 3.497 | <0.001 | 1.011 | 0.140 | 0.209 | 7.205 | <0.001 | 0.965 | 0.141 | 0.199 | 6.848 | <0.001 |
| Race | -0.010 | 0.011 | -0.033 | -0.922 | 0.357 | -0.163 | 0.080 | -0.059 | -2.024 | 0.043 | -0.156 | 0.080 | -0.056 | -1.944 | 0.052 |
| Education | 0.022 | 0.009 | 0.097 | 2.587 | 0.010 | 1.090 | 0.066 | 0.491 | 16.485 | <0.001 | 1.074 | 0.066 | 0.484 | 16.229 | <0.001 |
| No recreational activity | reference | reference | reference | reference | reference | reference | reference | reference | reference | reference | reference | reference | reference | reference | reference |
| Below guideline | 0.064 | 0.027 | 0.256 | 2.383 | 0.017 | 0.195 | 0.208 | 0.081 | 0.941 | 0.347 | 0.150 | 0.208 | 0.062 | 0.719 | 0.472 |
| Meet guideline | 0.105 | 0.028 | 0.419 | 3.718 | <0.001 | 0.777 | 0.218 | 0.320 | 3.563 | <0.001 | 0.701 | 0.219 | 0.289 | 3.199 | 0.001 |
| Exceed guideline | 0.095 | 0.026 | 0.376 | 3.590 | <0.001 | 0.516 | 0.202 | 0.213 | 2.548 | 0.011 | 0.448 | 0.204 | 0.185 | 2.204 | 0.028 |
| PEF% | NA | NA | NA | NA | NA | NA | NA | NA | NA | NA | 0.713 | 0.286 | 0.074 | 2.497 | 0.013 |
| Notes: Model 1, Activity→PEF%; Model 2, Activity→Global cognitive score; Model 3, Activity, PEF%→Global cognitive score. | | | | | | | | | | | | | | | |

| **eTable 3. Correlation Coefficient Matrix of Variables in people without pulmonary diseases.** | | | | | | | | |
| --- | --- | --- | --- | --- | --- | --- | --- | --- |
| Variables | 1 | 2 | 3 | 4 | 5 | 6 | 7 | 8 |
| 1.Age |  |  |  |  |  |  |  |  |
| 2.Gender | 0.003 |  |  |  |  |  |  |  |
| 3.Race | -0.03 | 0.02 |  |  |  |  |  |  |
| 4.Education | -0.11** | 0.05 | -0.08 |  |  |  |  |  |
| 5.PEF in percent predicted | 0.04 | 0.09* | 0.003 | 0.14*** |  |  |  |  |
| 6.Physical activity | -0.03 | -0.13** | -0.08 | 0.14*** | 0.13** |  |  |  |
| 7.Recreational activity | -0.03 | -0.08 | -0.07 | 0.24*** | 0.20*** | 0.68*** |  |  |
| 8.Work activity | -0.02 | -0.06 | -0.06 | 0.003 | 0.03 | 0.72*** | 0.19*** |  |
| 9.Global cognitive score | -0.26*** | 0.24*** | -0.20*** | 0.53*** | 0.19*** | 0.14*** | 0.22*** | 0.05 |
| Notes: *p <0 .05; **p <0 .01; ***p <0 .001 | | | | | | | | |

| **eTable 4. Comparison of the characteristics between participants included and excluded in this study** | | | |
| --- | --- | --- | --- |
| **Characteristic** | **Included** | **Excluded** | **P Value** |
|  | **(n=729)** | **(n=632)** |  |
| Age, mean (SD), y | 67.12(5.29) | 74.45(6.99) | <0.01 |
| Gender, weighted % |  |  | 0.80 |
| Male | 46.42 | 43.14 |  |
| Female | 53.58 | 56.86 |  |
| Race, weighted % |  |  | <0.01 |
| Mexican American | 3.01 | 3.8 |  |
| Non-Hispanic white | 87.12 | 86.1 |  |
| Non-Hispanic black | 9.87 | 10.1 |  |
| Education, weighted % |  |  | <0.01 |
| < High school | 14.04 | 26.25 |  |
| High school | 21.41 | 25 |  |
| Some college | 29.09 | 29.54 |  |
| College degree or more | 35.46 | 19.21 |  |
| BMI, mean (SD), kg/m^2^ | 29.42(6.23) | 27.75(6.19) | 0.34 |
| Smoking, weighted % |  |  | 0.41 |
| Never | 48.75 | 49.37 |  |
| Former | 38.68 | 41.83 |  |
| Current | 12.57 | 8.8 |  |
| Lung diesease, weighted % | 20.5 | 21.34 | 0.66 |
| High blood pressure, weighted % | 72.24 | 84.04 | 0.03 |
| Diabetes, weighted % | 17.56 | 20.21 | 0.62 |
| Cardiovascular diseases, weighted % | 10.33 | 23.18 | <0.01 |
| Stroke, weighted % | 3.92 | 18.62 | <0.01 |

**eReferences**

1. Hankinson JL, Odencrantz JR, Fedan KB: **Spirometric reference values from a sample of the general U.S. population**. *American journal of respiratory and critical care medicine* 1999, **159**(1):179-187.

2. (U.S.) NCfHS: **National Health and Nutrition Examination Survey: 2011-2012 Data Documentation, Codebook, and Frequencies**. <https://wwwn.cdc.gov/Nchs/Nhanes/2011-2012/SPX_G.htm>. Accessed December 2014.

3. Thorat YT, Salvi SS, Kodgule RR: **Peak flow meter with a questionnaire and mini-spirometer to help detect asthma and COPD in real-life clinical practice: a cross-sectional study**. *NPJ primary care respiratory medicine* 2017, **27**(1):32.

4. Jackson H, Hubbard R: **Detecting chronic obstructive pulmonary disease using peak flow rate: cross sectional survey**. *BMJ (Clinical research ed)* 2003, **327**(7416):653-654.

5. Fuller PM, Gooley JJ, Saper CB: **Neurobiology of the sleep-wake cycle: sleep architecture, circadian regulation, and regulatory feedback**. *Journal of biological rhythms* 2006, **21**(6):482-493.

6. de Feijter M, Kocevska D, Ikram MA, Luik AI: **The bidirectional association of 24-h activity rhythms and sleep with depressive symptoms in middle-aged and elderly persons**. *Psychological medicine* 2023, **53**(4):1418-1425.

7. Smagula SF, Ancoli-Israel S, Blackwell T, Boudreau R, Stefanick ML, Paudel ML, Stone KL, Cauley JA: **Circadian rest-activity rhythms predict future increases in depressive symptoms among community-dwelling older men**. *The American journal of geriatric psychiatry : official journal of the American Association for Geriatric Psychiatry* 2015, **23**(5):495-505.
